# From Symmetric Glycerol Derivatives to Dissymmetric Chlorohydrins

**DOI:** 10.3390/molecules23110000

**Published:** 2011-03-02

**Authors:** Carmen Solarte, Marc Escribà, Jordi Eras, Gemma Villorbina, Ramon Canela, Mercè Balcells

**Affiliations:** Department of Chemistry, Lleida University, Alcalde Rovira Roure 191, 25198 Lleida, Spain

**Keywords:** glycerol, chlorohydrin esters, dissymmetry, 1-butanol, 1,4-dioxane

## Abstract

The anticipated worldwide increase in biodiesel production will result in an accumulation of glycerol for which there are insufficient conventional uses. The surplus of this by-product has increased rapidly during the last decade, prompting a search for new glycerol applications. We describe here the synthesis of dissymmetric chlorohydrin esters from symmetric 1,3-dichloro-2-propyl esters obtained from glycerol. We studied the influence of two solvents: 1,4-dioxane and 1-butanol and two bases: sodium carbonate and 1-butylimidazole, on the synthesis of dissymmetric chlorohydrin esters. In addition, we studied the influence of other bases (potassium and lithium carbonates) in the reaction using 1,4-dioxane as the solvent. The highest yield was obtained using 1,4-dioxane and sodium carbonate.

## 1. Introduction

Glycerol (1,2,3-propanetriol), is one of the most versatile and valuable chemicals known to man. Glycerol has over 1,500 known end uses, including applications as an ingredient or processing aid in cosmetics, toiletries, personal care products, pharmaceutical formulations and food [1]. Traditionally, it is obtained as a by-product in four different processes: soap manufacture (saponification), fatty acid production (hydrolysis), fatty ester production (transesterification) and microbial fermentation. It can also be synthesized from propylene oxide [2].

Production of glycerol as a by-product has increased rapidly during the last decade as a consequence of biodiesel production [3,4]. Biodiesel is a common term for the different alkyl fatty esters formed by transesterification of vegetable oils or fats with an alcohol, usually methanol, with or without a catalyst. It has been implemented as an alternative to transportation fuels in Europe, the United States, Brazil and other countries as a result of increased interest in using renewable energy sources to reduce greenhouse gas emissions and to alleviate the depletion of fossil fuel reserves [5,6].

In the biodiesel process, glycerol is usually generated at the rate of one mole of glycerol for every three moles of synthesized methyl esters; approximately 10% of the total product by mass. Thus, the increase in biodiesel production will result in an accumulation of glycerol for which there is currently insufficient conventional uses. This situation has created a glut in the glycerol market and a drop in its price.

Crude glycerol generated during the transesterification process leading to biodiesel contains impurities such as methanol, water, inorganic salts (catalyst residue), free fatty acids, unreacted mono-, di- and triglycerides, methyl esters and a variety of other organic materials, depending on the biodiesel process [7,8], hence it must be purified before subsequent use in conventional applications.

All of the factors described above have prompted a search for new glycerol applications. Recent reviews have reported the utilization of glycerol to produce different value-added chemicals [2,3,4,9,10]. As a way to revalorize glycerol, our group has adopted new approaches based on the transformation of this polyol into halohydrin esters [11,12,13]. These products can be used as building blocks for the synthesis of a range of biologically active natural and synthetic products [14,15,16,17,18,19,20,21,22,23,24,25,26,27].

In this paper, we report the results obtained when two solvents (1,4-dioxane and 1-butanol) and different bases are used to prepare dissymmetric chlorohydrin esters from symmetric 1,3-dichloro-2-propyl esters obtained from glycerol.

## 2. Results and Discussion

Initially, we studied the influence of combining two solvents and two bases in the synthesis of dissymmetric chlorohydrin esters. Thus, several 1,3-dichloro-2-propyl esters **1a–d** were treated with water and an organic (1-butylimidazole) or inorganic (sodium carbonate) base in two different solvents: 1,4-dioxane or 1-butanol (Table 1). Most of the esters used were α,α-dimethyl-substituted (versatic) esters. Yields of the reactions carried out with sodium carbonate and 1,4-dioxane were usually higher than the corresponding reactions with sodium carbonate and 1-butanol, except in the reaction with 2-chloro-1-(chloromethyl)ethyl 2,2-dimethylpropionate (**1b**), where the yield was the same. A similar tendency was observed using 1-butylimidazole as the base, but this time the exception was in the reaction with 2-chloro-1-(chloromethyl)ethyl 2-methyl-2-phenylpropionate (**1d**).

In the reactions with 2-chloro-1-(chloromethyl)ethyl hexadecanoate (**1a**), the best solvent was 1,4-dioxane. There was no difference between the use of either sodium carbonate or 1-butylimidazole. The product, **2a**, was not observed when 1-butanol was used. Probably, because this not an α,α-dimethyl-substituted ester, and it is not stable in such a solvent.

In the reactions with 2-chloro-1-(chloromethyl)ethyl 2,2-dimethylpropionate (**1b**), both solvents (1,4-dioxane and 1-butanol) with sodium carbonate and 1,4-dioxane with 1-butylimidazole produced the highest yields.

Finally, the combination 1,4-dioxane/sodium carbonate and 1-butanol/1-butylimidazole gave the highest yields with the other compounds (**2c–d**) when the bases were compared in the same solvent. These results can be explained by considering the high stability of the hydrolytic processes of the α,α-dimethyl-substituted esters. 

**Scheme 1:**
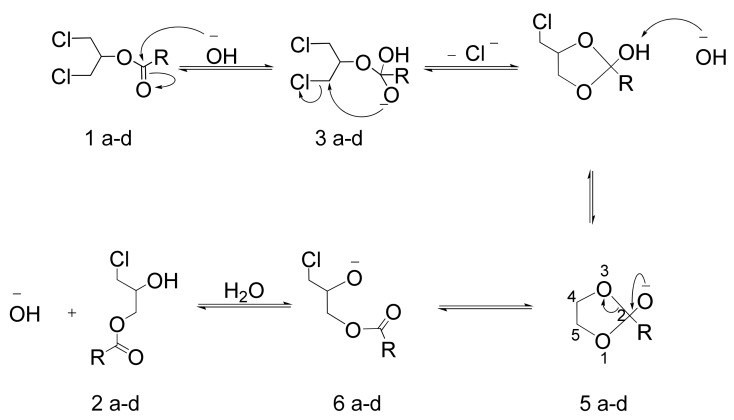
Mechanism proposed to explain the formation of 3-chloro-2-hydroxy-1-propyl esters using water and base as reagent.

We propose the mechanism shown in Scheme 1 for the formation of 3-chloro-2-hydroxy-1-propyl esters. The transposition process observed here has already been described by our group in the preparation of glycidyl palmitate [13]. This mechanism is compatible with the experimental results. Similar behaviour was described by Leggetter and Brown studying the influence of substituents on the opening of substituted 1,3-dioxolanes [28,29]. Therefore, the preferred cleavage of C2-O3 bond might be explained by electron-withdrawing substituents, which should produce on C4 a higher positive charge density than on C5. This will provoke the formation of the indicated chlorohydrin ester instead of the other putative regioisomer.

In addition, we studied the influence of other bases using 1,4-dioxane as the solvent because it had been the best solvent considering all the tested compounds. Our aim was to improve the obtained yields. Two esters were chosen as model compounds. One was the palmitic ester because it does not possess α,α-dimethyl-substitution. The other was α,α-dimethylphenyl acetate. Considering that there was a small difference in the yield of compounds **2a** and **2d** when organic or inorganic bases were used in 1,4-dioxane, the effect of sodium, potassium and lithium carbonates on 3-chloro-2-hydroxy-1-propyl ester formation was checked.

Satisfactory yields were obtained using a cheap base such as sodium carbonate (Table 2), independent of the acyl compound used. When the effect of each base on the transformation of dichloro esters **1a** and **1d** was studied, the percentages of 3-chloro-2-hydroxy-1-propyl esters **2a** and **2d** were always higher in the transformation of 2-chloro-1-(chloromethyl)ethyl hexadecanoate (entry a) than in the transformation of phenyl acetate derivatives, irrespective of the base used. 

The yield differences with the inorganic bases may be explained by differences in water solubility of these salts. Sodium and potassium carbonate solubility increases with increasing temperature and therefore the availability of hydroxyl groups which act as nucleophile. On the other hand, potassium cations are more basic than sodium cations. Yields in the reactions with potassium carbonate are lower because 3-chloro-2-hydroxy-1-propyl ester was transformed into an epoxide (result not shown). These compounds were not observed when sodium carbonate was used. Lithium carbonate has behaves differently because its solubility decreases with increasing temperature.

Finally, a multigram-scale synthesis of 3-chloro-2-hydroxy-1-propyl 2,2-dimethylpropanoate **2b** was carried out, considering that the best yields were always obtained using the 2,2-dimethylpropanoate ester **1b**. 1,4-Dioxane and sodium carbonate were used to transform 5.05 g of **1b** into compound **2b**. A yield of 2.9 g of **2b** (62% yield) was obtained after purification by distillation, showing that the method is fully applicable to obtain gram quantities of dissymmetric chlorohydrins from the corresponding symmetric esters.

## 3. Experimental 

### 3.1. General 

NMR (400/100 MHz) spectra were recorded on a Varian 400 spectrometer using CDCl_3_ as solvent, as indicated. Chemical shifts are reported in ppm (δ) relative to the TMS signal. High-resolution mass spectral (HRMS) data were obtained by direct infusion on Agilent G6510AA Q-TOF MS using electrospray ionization source (ESI). IR spectra were recorded on a Magna IR 560 Nicolet FTIR spectrophotometer in the range 4000–600 cm^−1^ with KBr pellets. Spectra dates are reported in reciprocal centimetres (cm^−1^). Palmitic acid (98%), 2,2-dimethylbutyric acid (96%), trimethylacetic acid (99%) and butylimidazole (98%) were purchased from Aldrich. Potassium carbonate (99%) and lithium carbonate (98%) were obtained from Fluka. α,α-Dimethylphenylacetic acid was purchased from Alfa Aesar. Sodium carbonate (98%) was obtained from Panreac. Solvents and reagents were dried using conventional methods prior to use.

### 3.2. Procedure for the syntheses of 2-chloro-1-(chloromethyl)ethyl esters ***1***

Carboxylic acid (1 mmol), glycerol (184 mg, 2 mmol) and chlorotrimethylsilane (540 mg, 5 mmol) were added to a reaction vial fitted with a PTFE-lined cap. The mixture was heated at 80 °C for 48 h. After cooling, an organic solvent was added, and the mixture was washed three times with water. The organic layer was dried over anhydrous MgSO_4_, and the solvent was evaporated under vacuum. The residue was purified by dry flash column chromatography on silica gel. 

*2-Chloro-1-(chloromethyl)ethyl hexadecanoate* (**1a**; CAS: 72165-62-9). ^1^H-NMR δ: 0.88 (t, *J* = 6.4 Hz, 3 H), 1.20–1.40 (m, 24 H) 1.60–1.75 (m, 2 H), 2.37 (t, *J* = 7.5 Hz, 2 H) 3.72 (dd, *J* = 11.6, 5.1 Hz, 2 H) 3.77 (dd, *J* = 11.6, 5.3 Hz, 2 H) 5.18, (tt, *J* = 5.1, 5.3 Hz, 1 H). ^13^C-NMR δ: 14.1, 22.7, 24.8, 29.0, 29.2, 29.3, 29.4, 29.6, 29.7, 31.9, 34.1, 42.4, 71.4,172.7. MS *m/z*: 368 (M+1)^+^, 366 (M-1) ^+^, 239, 43 [12,30].

*2-Chloro-1-(chloromethyl)ethyl 2,2-dimethylpropanoate* (**1b**; CAS: 220499-01-4). ^1^H-NMR δ: 5.21 (m, 1H, C*H*), 3.83 (m, 4H, C*H_2_*-Cl), 1.24 (s, 9H, 3C*H_3_*). ^13^C-NMR δ: 177.3 (*C*=O), 43.0 (*C*H_2_-Cl), 39.0 ((CH_3_)_2_-*C*), 27.0 (3*C*H_3_) [12,31]. 

*2-Chloro-1-(chloromethyl)ethyl 2,2-dimethylbutanoate* (**1c**). ^1^H-NMR δ: 5.14 (quin, *J* = 5.2 Hz, 2H, O-C*H*_2_), 3.72 (dd, *J*_1_ = 5.2 Hz, *J*_2_ = 2.3 Hz, 4H, 2 C*H*_2_-Cl), 1.59 (q, *J* = 7.5 Hz, 2H, C*H*_2_-CH_3_), 1.17 (s, 6H, (C*H*_3_)_2_-C), 0.85 (t, *J* = 7.5 Hz, 3H, CH_2_-C*H*_3_). ^13^C-NMR δ: 176.8 (*C*=O), 71.4 (O-*C*H), 42.9 ((CH_3_)_2_-*C*), 42.5 (*C*H_2_-Cl), 33.1 (*C*H_2_-CH_3_), 24.5 ((*C*H_3_)_2_-C), 9.2 (CH_2_-*C*H_3_). IR (KBr) ν_max_: 2967.4, 2937.2, 2876.8, 1729.0, 1237.1, 1137.9 cm^−1^. HRMS (ESI+) [M+Na]^+^ C_9_H_16_Cl_2_O_2_Na calculated: 249.0425, found: 249.0518.

*2-Chloro-1-(chloromethyl)ethyl-2-methyl-2-phenylpropanoate* **(1d)**. ^1^H-NMR δ: 7.46–7.21, m, 5H, C*H*_ar_), 5.16 (quin, *J* = 5.3 Hz, 2H, O-C*H*_2_), 3.73–3.59 (m, 4H, 2 C*H*_2_-Cl), 1.63 (s, 6H, (C*H*_3_)_2_-C). ^13^C- NMR δ: 175.6 (*C*=O), 143.8 (*C*_ar_), 128.4, 126.9, 125.7 (*C*H_ar_), 72.0 (O-*C*H), 46.7 ((CH_3_)_2_-*C*), 42.3 (*C*H_2_-Cl), 26.3 ((*C*H_3_)_2_-C). IR (KBr) ν_max_: 3083.9, 3053.7, 3023.5, 2976.1, 2928.6, 2872.5, 1729.0, 1241.4, 1133.5 cm^−1^. HRMS (ESI+) [M+Na]^+^ C_13_H_16_Cl_2_O_2_Na calculated: 297.0425, found: 297.0531.

### 3.3. Procedure for the syntheses of 3-chloro-2-hydroxy-1-propyl esters ***2***

A solution of 2-chloro-1-(chloromethyl)ethyl ester (1 mmol), water (29 μL, 1.6 mmol) and either sodium carbonate (106 mg, 1 mmol) or 1-butylimidazole (124 mg, 1 mmol) in dried solvent (1,4-dioxane or 1-butanol (3 mL)) was heated at 115 °C for 48 h in a capped reaction vial. After cooling, dichloromethane was added, and the mixture was washed three times with water. The organic layer was dried over anhydrous MgSO_4_, and the solvent was evaporated. The crude compound was analyzed by ^1^H-NMR to determine the yield of the corresponding product using *N,N*-dimethylformamide (DMF) as an internal standard. Further purification by dry flash column chromatography on silica gel (hexane/ethyl acetate) gave the desired product.

*3-Chloro-2-hydroxy-1-propyl hexadecanoate* (**2a**; CAS: 30557-04-1). ^1^H-NMR δ: 0.90 (t, *J* = 6.3 Hz, 3H, C*H_3_*), 1.20–1.40 (m, 24H, (C*H_2_*)*_12_*), 1.66 (m, 2H, C*H_2_*-CH_2_COO), 2.30 (t, *J* = 7.5 Hz, 2H, C*H_2_*-COO), 3.59 and 3.65 (dd, *J* = 8.5 Hz, *J* = 5.2 Hz, 2H, C*H_2_*-Cl), 4.00 (quin, *J* = 5.2 Hz, 1H, C*H*-OH), 4.15 (dd, *J* = 5.2 Hz, *J* = 1.7 Hz, 2H, O-C*H_2_*-CH). ^13^C-NMR δ: 177 (*C*=O), 70 (*C*H-OH), 66 (O-*C*H_2_), 45 (*C*H_2_-Cl), 34 (*C*H_2_-COO), 32–22 (13C, *C*H_2_), 17 (*C*H_3_). GC-MS m/z: 348 [M]^+^, 299 [M-CH_2_Cl]^+^, 269 [M-C_2_H_2_ClO]^+^, 255 [M-C_3_H_6_ClO]^+^, 239 [M-C_3_H_6_ClO_2_]^+^, 152 [C_5_H_9_O_3_Cl]^+^ (McLafferty rearrangement) [13].

*3-Chloro-2-hydroxy-1-propyl 2,2-dimethylpropanoate* (**2b**; CAS: 52562-21-7). ^1^H-NMR δ: 4.05 (dd, *J*_1_ = 5.68 Hz, *J*_2_ = 1.26 Hz, 2H, O-C*H_2_*), 3.90 (m, 1H, C*H*-OH), 3.45 (m, 2H, C*H_2_*-Cl), 1.04 (s, 9H, 3C*H_3_*). ^13^C-NMR δ: 179.5 (*C*=O), 70 (*C*H-OH), 65.5 (O-*C*H_2_), 46 (*C*H_2_-Cl), 39 ((CH_3_)_2_-*C*), 27.3 (3*C*H_3_). IR ATR ν_max_.: 3437.5, 2953, 1718, 1500, 1296, 1156, 1046, 765, 703, 640 cm^−1^. HRMS (ESI+) [M+Na]^+^ C_8_H_15_ClO_3_Na calculated: 217.0607, found: 217.0701.

*3-Chloro-2-hydroxy-1-propyl 2,2-dimethylbutanoate* (**2c**). ^1^H-NMR δ: 4.20 (dd, *J*_1_ = 5.9 Hz, *J*_2_ = 1.2 Hz, 2H, O-C*H*_2_), 4.05 (quin, *J* = 5.3 Hz, 1H, C*H*-OH), 3.65–3.54 (m, 2H, C*H_2_*-Cl), 1.56 (q, *J* = 7.5 Hz, 2H, C*H*_2_-CH_3_), 1.16 (s, 6H, (C*H*_3_)_2_-C), 0.83 (t, *J* = 7.5 Hz, 3H, CH_2_-C*H*_3_). ^13^C-NMR δ: 178.1 (*C*=O), 69.8 (*C*H-OH), 65.0 (O-*C*H_2_), 45.9 (*C*H_2_-Cl), 42.8 ((CH_3_)_2_-*C*), 33.3 (*C*H_2_-CH_3_), 24.6 ((*C*H_3_)_2_-C), 9.2 (CH_2_-*C*H_3_). IR (KBr) ν_max_: 3480.9, 2967.4, 2937.2, 2876.8, 1724.7, 1241.4, 1150.8 cm^−1^. HRMS (ESI+) [M+Na]^+^ C_9_H_17_ClO_3_Na calculated: 231.0764, found: 231.0860.

*3-Chloro-2-hydroxy-1-propyl 2-methyl-2-phenylpropanoate* (**2d**). ^1^H-NMR δ: 7.39–7.23 (m, 5H, C*H*_ar_), 4.21 (dd, *J*_1_ = 5.2 Hz, *J*_2_ = 2.0 Hz, 2H, O-C*H*_2_), 3.97 (quin, *J* = 5.3 Hz, 1H, C*H*-OH), 3.46–3.36 (m, 2H, C*H*_2_-Cl), 1.62 (s, 6H, (C*H*_3_)_2_-C). ^13^C-NMR δ: 176.8 (*C*=O) 144.2 (*C*_ar_), 128.5, 126.9, 125.5 (*C*H_ar_), 69.5 (*C*H-OH), 65.2 (O-*C*H_2_), 46.6 ((CH_3_)_2_-*C*), 45.7 (*C*H_2_-Cl), 26.3 ((*C*H_3_)_2_-C). IR (KBr) ν_max_: 3480.9, 3083.9, 3053.7, 3019.2, 2971.7, 2924.3, 1729.0, 1245.7, 1142.2 cm^−1^. HRMS (ESI+) [M+Na]^+^ C_13_H_17_ClO_3_Na calculated: 279.0764, found: 279.0873. 

### 3.4. Procedure for the syntheses of 3-chloro-2-hydroxy-1-propyl esters ***2a*** and ***2d*** using different inorganic bases 

A solution of 2-chloro-1-(chloromethyl)ethyl ester (1 mmol), water (29 μL, 1.6 mmol) and a carbonate (1 mmol) in dried 1,4-dioxane (3 mL) was heated at 115 °C for 48 h in a capped reaction vial. After cooling, dichloromethane was added and the mixture was washed three times with water. The organic layer was dried over anhydrous MgSO_4_, and the solvent was distilled. The crude compound was analyzed by ^1^H-NMR to determine the yield of the corresponding product using DMF as an internal standard. Further purification by dry flash column chromatography on silica gel (hexane/ethyl acetate) gave the desired product.

### 3.5. Procedure for the multigram-scale syntheses of 3-chloro-2-hydroxy-1-propyl 2,2-dimethylpropanoate *(**2b**)*

A solution of 2-chloro-1-(chloromethyl)ethyl 2,2-dimethylpropanoate (5.08 g, 24 mmol), water (0.7 mL, 38.4 mmol) and sodium carbonate (2.54 g, 24 mmol) in dried 1,4-dioxane (72 mL) was heated at 115 °C for 48 h in a capped reactor. After cooling, dichloromethane was added and the mixture was washed three times with water. The organic layer was dried over anhydrous MgSO_4_, and the mixture was distilled obtaining 2.9 g of the desired compound (62% yield) b.p.: 70 °C/ 40 Pa. The compound was identified by ^1^H-NMR and ^13^C-NMR.

## 4. Conclusions 

1,3-Dichloro-2-propyl esters prepared from glycerol can be transformed into the corresponding 3-chloro-2-hydroxy-1-propyl esters. These dissymmetric chlorohydrin esters are potentially valuable compounds and can be used as building blocks. With the methodology described above, different 3-chloro-2-hydroxy-1-propyl esters were synthesized with all studied compounds; better reaction conditions were obtained when using sodium carbonate as base and 1,4-dioxane as solvent. In addition, when different inorganic carbonates were used, sodium carbonate gave the best results. The best procedure developed allows to prepare 3-chloro-2-hydroxy-1-propyl 2,2-dimethylpropanoate on a multigram-scale.

## Figures and Tables

**Table 1:**
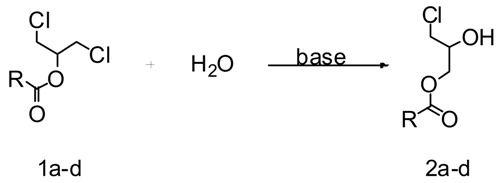
Synthesis of 3-chloro-2-hydroxy-1-propyl esters using various solvents and bases.

Entry	R	Solvent	Base	Yield^a^ of 2 (%)
a	CH_3_(CH_2_)_14_–	1,4-Dioxane	Sodium carbonate	48
a	CH_3_(CH_2_)_14_–	1,4-Dioxane	1-Butylimidazole	49
a	CH_3_(CH_2_)_14_–	1-Butanol	Sodium carbonate	–
a	CH_3_(CH_2_)_14_–	1-Butanol	1-Butylimidazole	–
b	(CH_3_)_3_C–	1,4-Dioxane	Sodium carbonate	62
b	(CH_3_)_3_C–	1,4-Dioxane	1-Butylimidazole	61
b	(CH_3_)_3_C–	1-Butanol	Sodium carbonate	62
b	(CH_3_)_3_C–	1-Butanol	1-Butylimidazole	45
c	CH_3_CH_2_(CH_3_)_2_C–	1,4-Dioxane	Sodium carbonate	36
c	CH_3_CH_2_(CH_3_)_2_C–	1,4-Dioxane	1-Butylimidazole	30
c	CH_3_CH_2_(CH_3_)_2_C–	1-Butanol	Sodium carbonate	–
c	CH_3_CH_2_(CH_3_)_2_C–	1-Butanol	1-Butylimidazole	23
d	Ph(CH_3_)_2_C–	1,4-Dioxane	Sodium carbonate	35
d	Ph(CH_3_)_2_C–	1,4-Dioxane	1-Butylimidazole	33
d	Ph(CH_3_)_2_C–	1-Butanol	Sodium carbonate	7
d	Ph(CH_3_)_2_C–	1-Butanol	1-Butylimidazole	33

^a^ Yield calculated by ^1^H-NMR using *N*,*N*-dimethylformamide as internal standard.

**Table 2:**
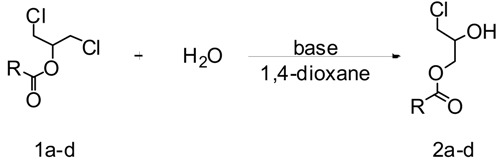
Influence of different inorganic bases on 3-chloro-2-hydroxy-1-propyl ester formation in 1,4-dioxane.

Entry	R	Base	Yield^a^ of 2 (%)
a	CH_3_(CH_2_)_14_–	Sodium carbonate	48
a	CH_3_(CH_2_)_14_–	Potassium carbonate	25
a	CH_3_(CH_2_)_14_–	Lithium carbonate	35
d	Ph(CH_3_)_2_C–	Sodium carbonate	35
d	Ph(CH_3_)_2_C–	Potassium carbonate	23
d	Ph(CH_3_)_2_C–	Lithium carbonate	27
